# Supplementary material for: Synthesis of Novel Diketopyrrolopyrrole-Rhodamine Conjugates and Their Ability for Sensing Cu2+ and Li+
Source: Molecules. 2022 Oct 25;27(21):7219. doi: 10.3390/molecules27217219 (PMC9656197; doi:10.3390/molecules27217219)
Supplement: Supplementary file 1 [file molecules-27-07219-s001.zip › molecules-1977124-supplementary.pdf]

Supporting Information

Synthesis of Novel Diketopyrrolopyrrole-Rhodamine Conjugates and Their Ability for Sensing Cu^{2+} and Li^{+}

Carla Queirós [1,†], Vítor A. S. Almodôvar [2,†], Fábio Martins [1], Andreia Leite [1], Augusto C. Tomé [2] and Ana M. G. Silva [1,*]

[1] LAQV-REQUIMTE, Department of Chemistry and Biochemistry, University of Porto, 4169-007 Porto, Portugal
[2] LAQV-REQUIMTE, Department of Chemistry, University of Aveiro, 3810-193 Aveiro, Portugal
* Correspondence: ana.silva@fc.up.pt; +351-2-2040-2585
† These authors contributed equally.

Content

^{1}H NMR and ^{13}C NMR of chemosensors 1 and 2 2

MS of chemosensors 1 and 2 4

Spectroscopic properties of precursors $DPP(CHO)_2$ and RhoHyd 5

UV-Vis and fluorescence: solution studies for 1 and 2 towards anions and cations 6

UV-Vis and fluorescence: solution studies for $DPP(CHO)_2$ and RhoHyd towards anions and cations 7

Absorbance and fluorescence measurements of chemosensor 2 in the presence of Cu^{2+} 8

Absorbance and fluorescence measurements of chemosensors 1+Cu^{2+} and 2+Cu^{2+} in the presence of Li^{+} 9

Absorbance and fluorescence measurements of chemosensor 2+Cu^{2+} in the presence of $Li(CH_3COO)$ and LiBr salts 10

Absorbance and fluorescence measurements of chemosensors 1+Cu^{2+} and 2+Cu^{2+} in the presence of CN^{-} 10

Stern-Volmer graphical representations of chemosensor 1+Cu^{2+} in the presence of CN^{-} 11

^{1}H NMR and ^{13}C NMR of chemosensors 1 and 2

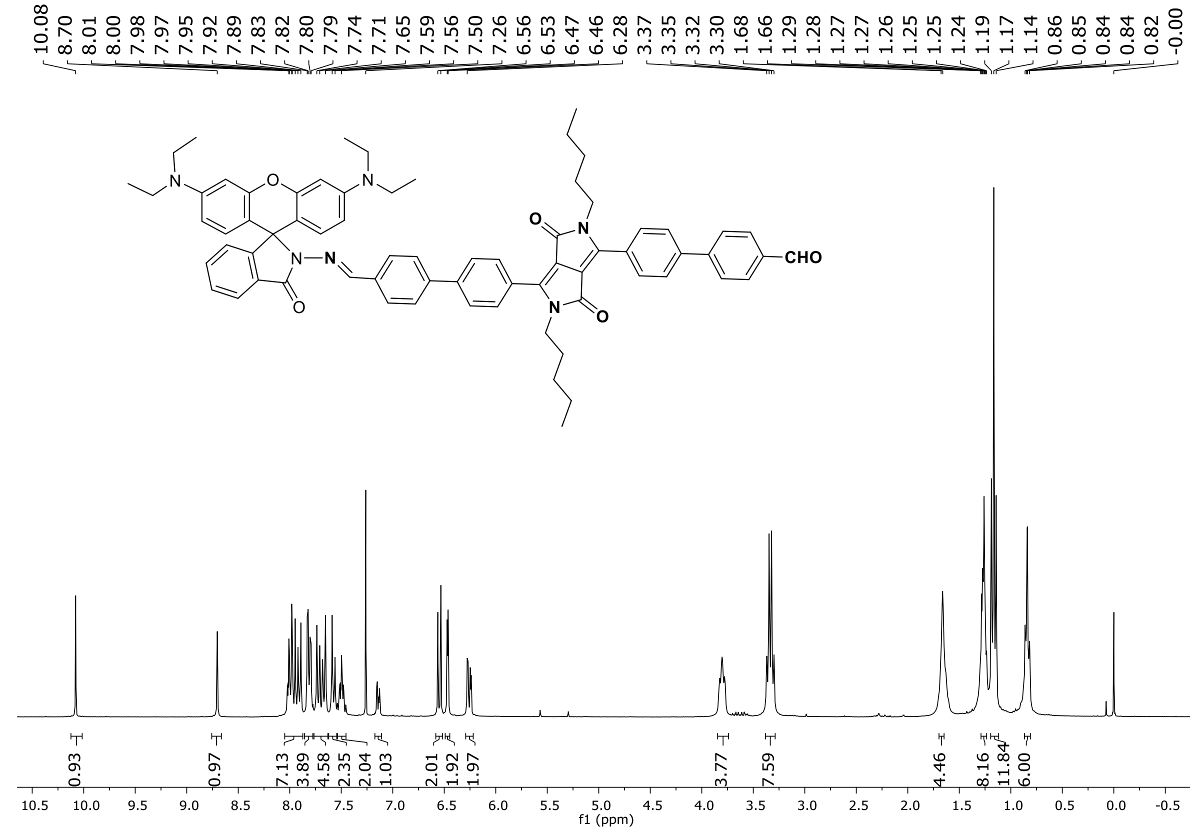

Figure S1. ^{1}H NMR spectrum of chemosensor **1**.

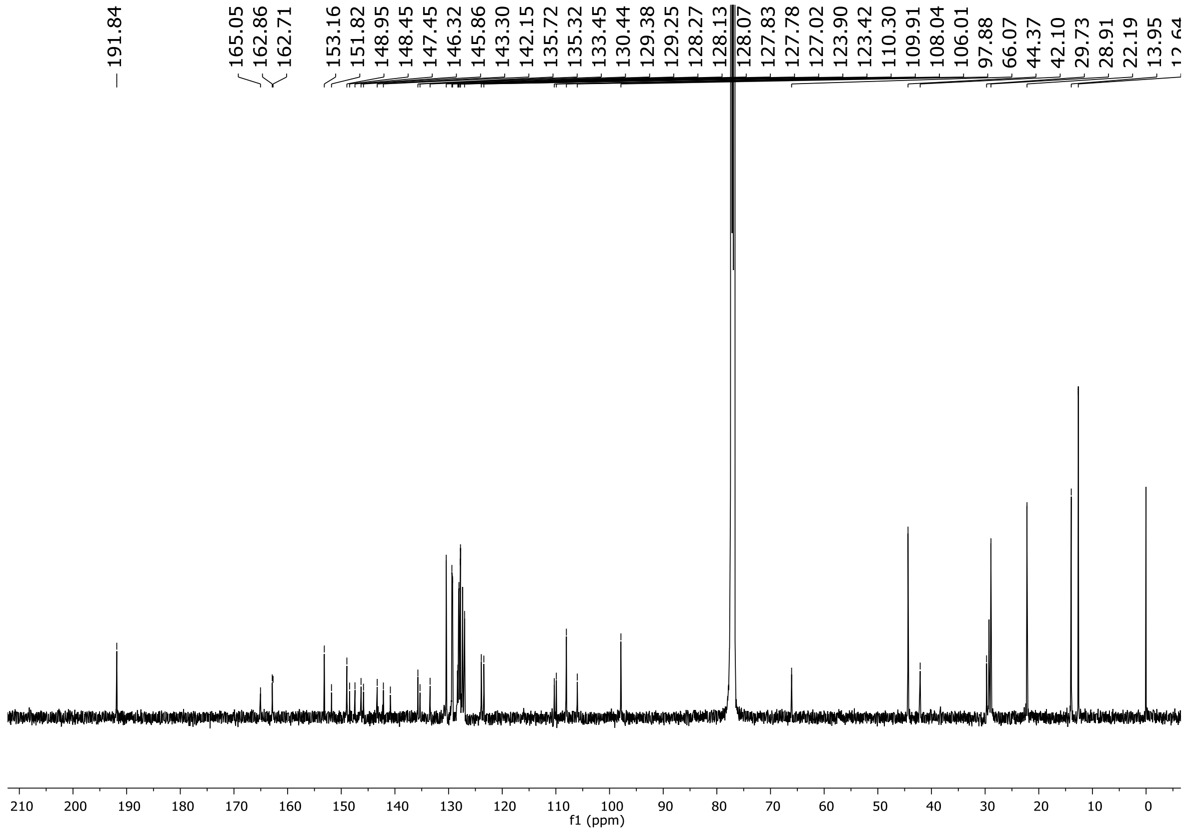

Figure S2. ^{13}C NMR spectrum of chemosensor **1**.

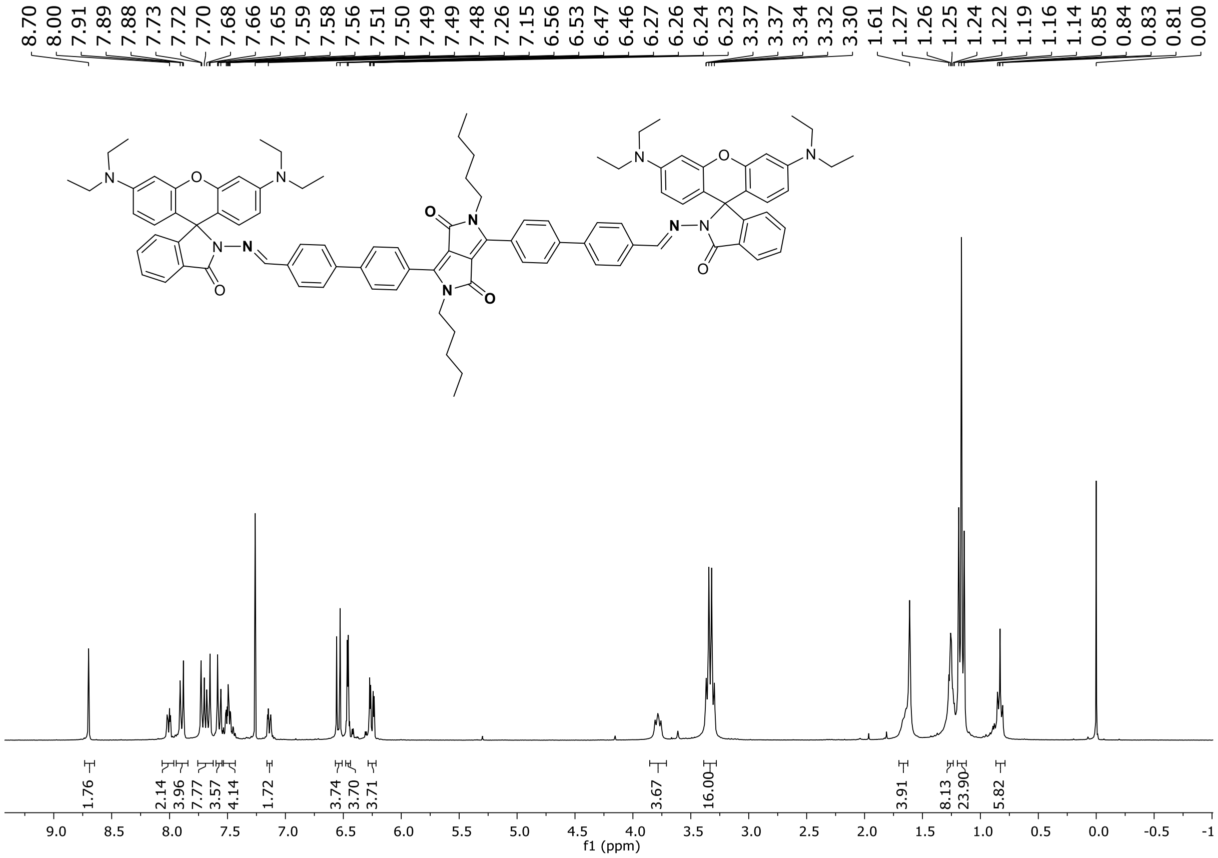

Figure S3. ^{1}H NMR spectrum of chemosensor **2**.

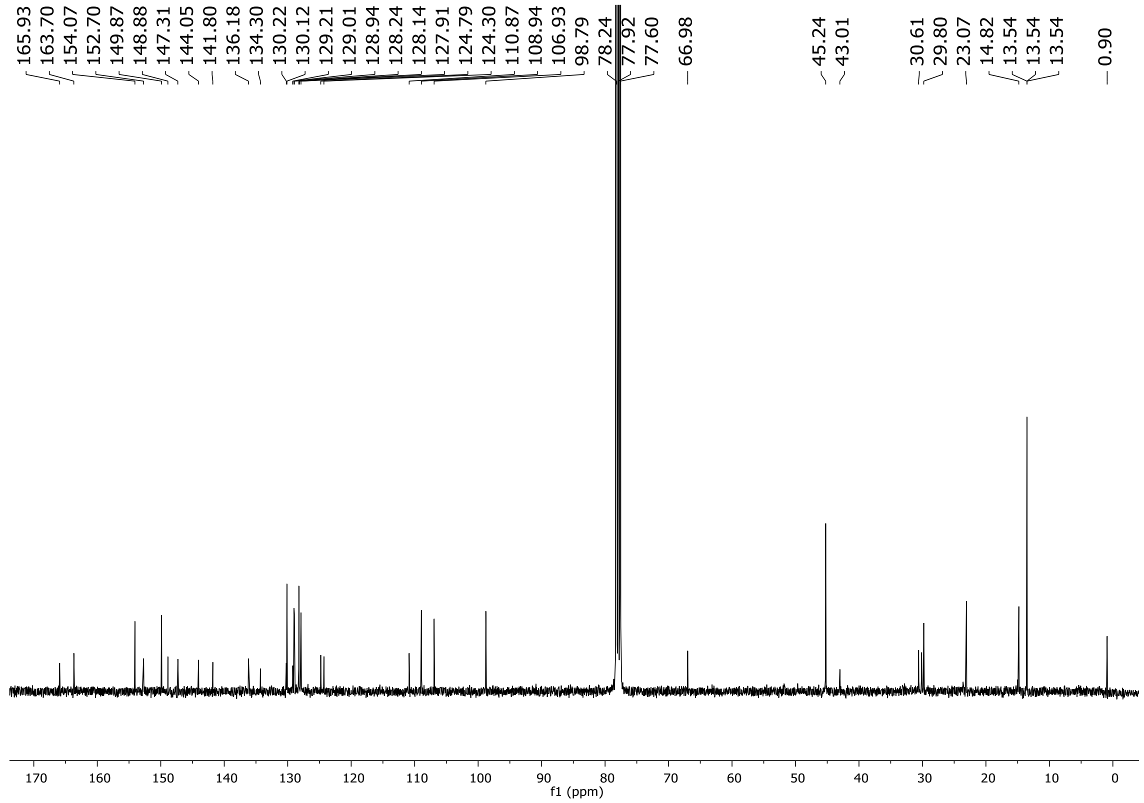

Figure S4. ^{13}C NMR spectrum of chemosensor **2**.

MS of chemosensors 1 and 2

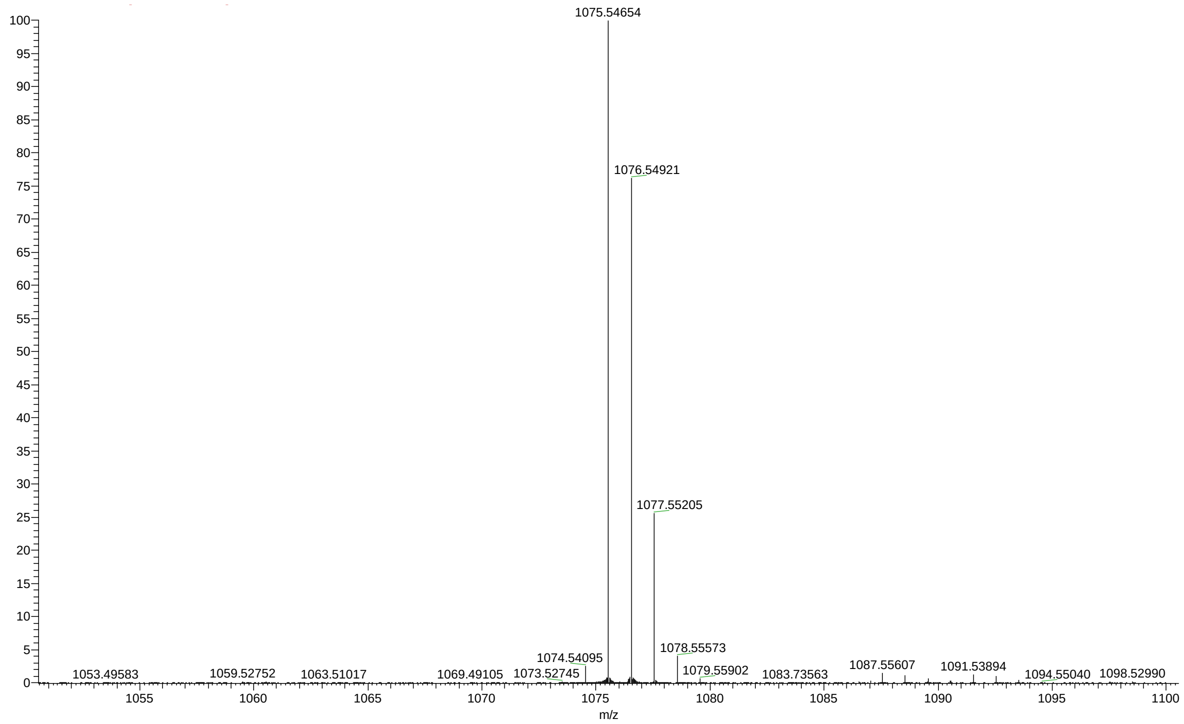

Figure S5. MS spectrum of chemosensor **1**.

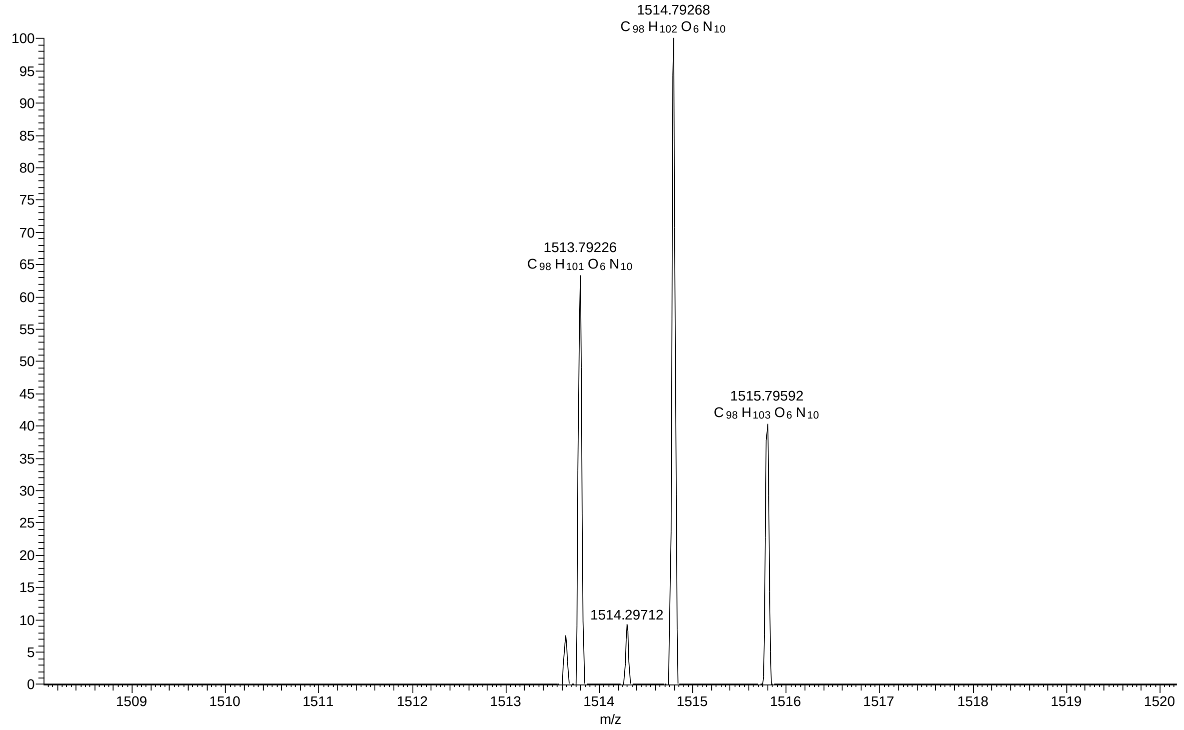

Figure S6. MS spectrum of chemosensor **2**.

Spectroscopic properties of precursors $DPP(CHO)_2$ and RhoHyd

Table S1. Spectroscopic properties (λ_{abs}, λ_{em}, molar absorptivity (ε), Stokes shift, fluorescence quantum yield (Φ_F) and lifetime (τ) of the precursors in CH_2Cl_2, EtOH and CH_3CN at 25 °C. .

	**λ_{abs} / nm ($\varepsilon \times 10^4$ / $dm^3mol^{-1}cm^{-1}$)**	**λ_{em} / nm**	**Stokes Shift / nm**	**Φ_F**	**τ / ns**
		**$DPP(CHO)_2$**			
CH_2Cl_2	268 (2.39), 323 (4.94), 492 (2.28)	573	80	0.92	4.8 ± 0.6
EtOH	316 (7.47), 485 (3.49)	566	81	0.88	Non determined
CH_3CN	265 (3.62), 318 (7.50), 485 (3.62)	568	83	0.88	Non determined
**RhoHyd**					
CH_2Cl_2	275 (4.39), 316 (1.80)	-	-	-	-
EtOH	273 (4.12), 311 (1.53)	-	-	-	-
CH_3CN	273 (1.50), 313 (1.41)	-	-	-	-

UV-Vis and fluorescence: solution studies for 1 and 2 towards anions and cations

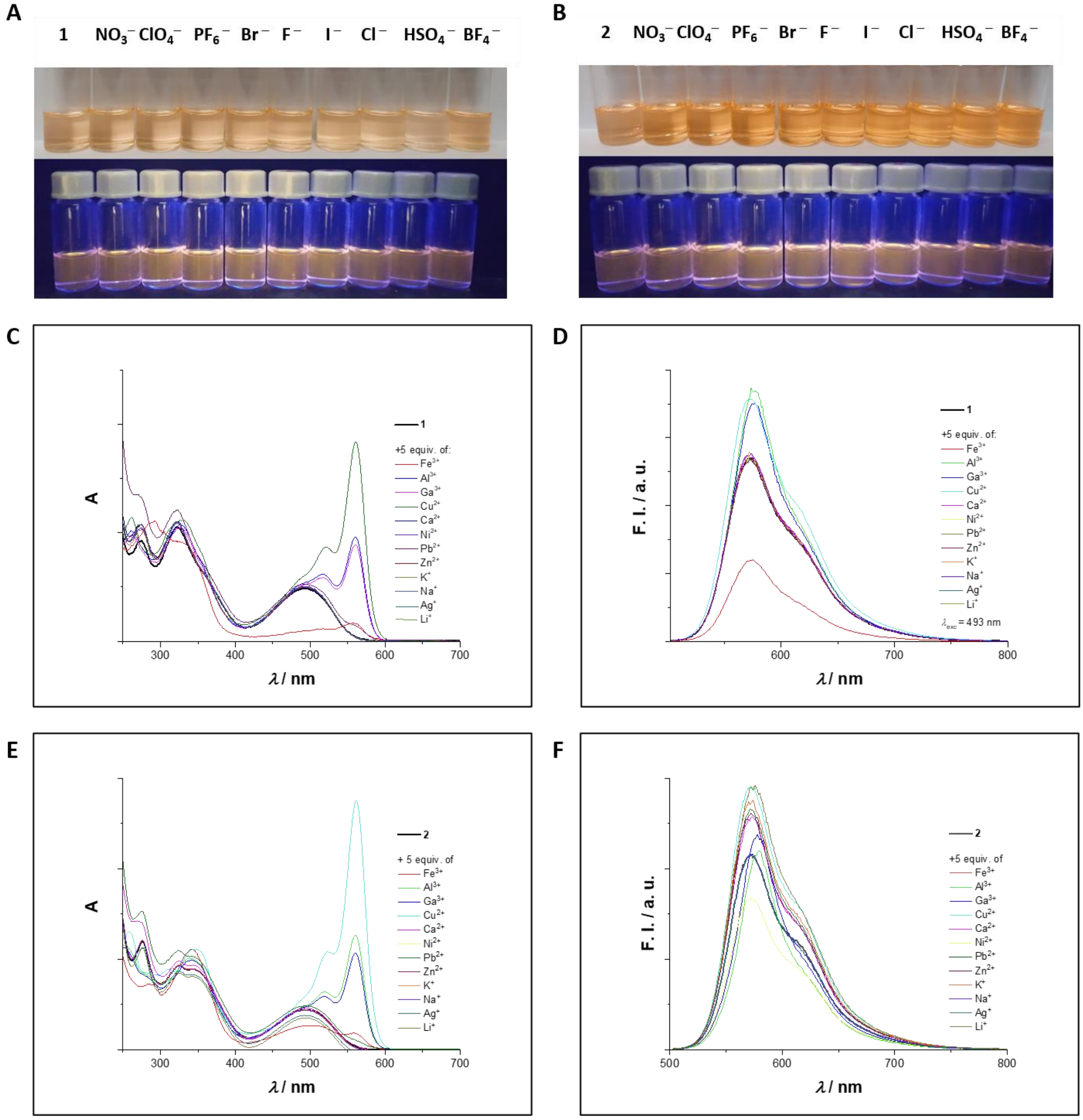

Figure S7. Photographs (up: visible and down: 354 nm lamp) of **1** (**A**) and **2** (**B**), in CH_2Cl_2, in the presence of 5 equiv. of anions (NO_3^-, ClO_4^-, PF_6^-, Br^-, F^-, I^-, Cl^-, HSO_4^- and BF_4^-) and absorption and fluorescence spectra of **1** (**C** and **D**) and **2** (**E** and **F**), in CH_2Cl_2, in the presence of 5 equiv. of cations (Fe^{3+}, Al^{3+}, Ga^{3+}, Cu^{2+}, Ca^{2+}, Ni^{2+}, Pb^{2+}, Zn^{2+}, K^+, Na^+, Ag^+ and Li^+).

UV-Vis and fluorescence: solution studies for DPP(CHO)2 and RhoHyd towards anions and cations

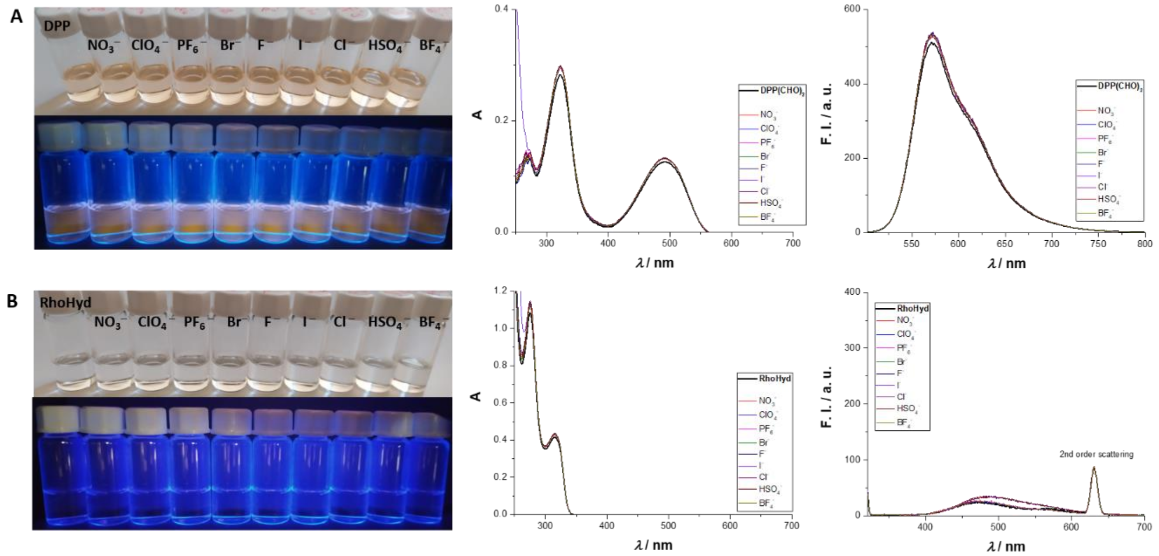

Figure S8. Photographs (up: visible and down: under a 354 nm lamp) and absorption and fluorescence spectra of **DPP(CHO)2** (**A**) and **RhoHyd** (**B**), in CH_2Cl_2, in the presence of 5 equiv. of anions (NO_3^-, ClO_4^-, PF_6^-, Br^-, F^-, I^-, Cl^-, HSO_4^- and BF_4^-).

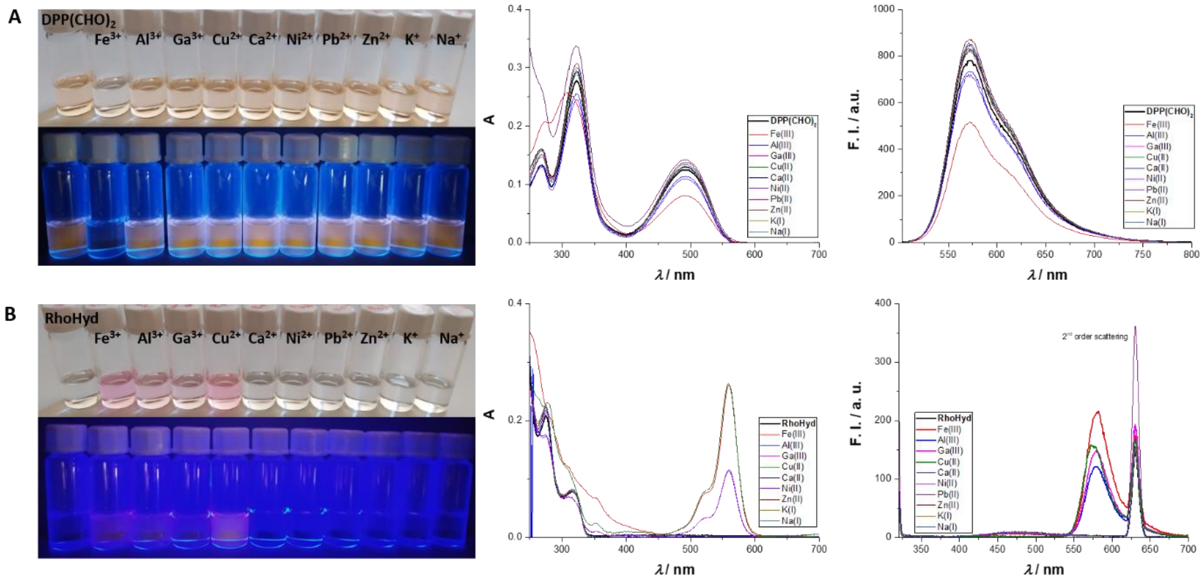

Figure S9. Photographs (up: visible and down: under a 354 nm lamp) and absorption and fluorescence spectra of of **DPP(CHO)2** (**A**) and **RhoHyd** (**B**), in CH_2Cl_2, in the presence of 5 equiv. of cations (Fe^{3+}, Al^{3+}, Ga^{3+}, Cu^{2+}, Ca^{2+}, Ni^{2+}, Pb^{2+}, Zn^{2+}, K^+ and Na^+).

Absorbance and fluorescence measurements of chemosensor 2 in the presence of Cu^{2+}

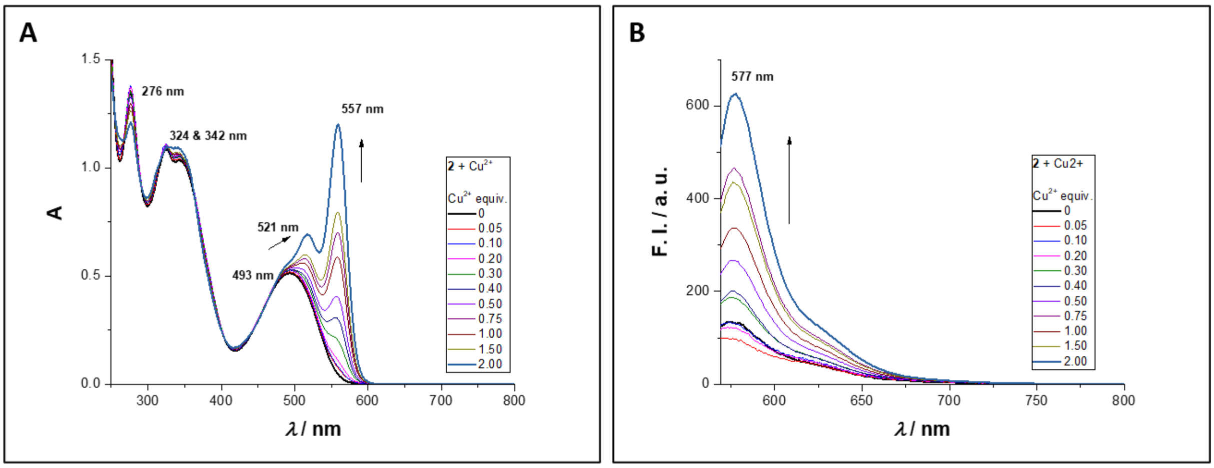

Figure S10. Absorbance (**A**) and fluorescence (**B**, using λ_{exc} = 557 nm) spectra of chemosensor **2** with increasing concentrations of Cu^{2+} (up to 2 equiv.).

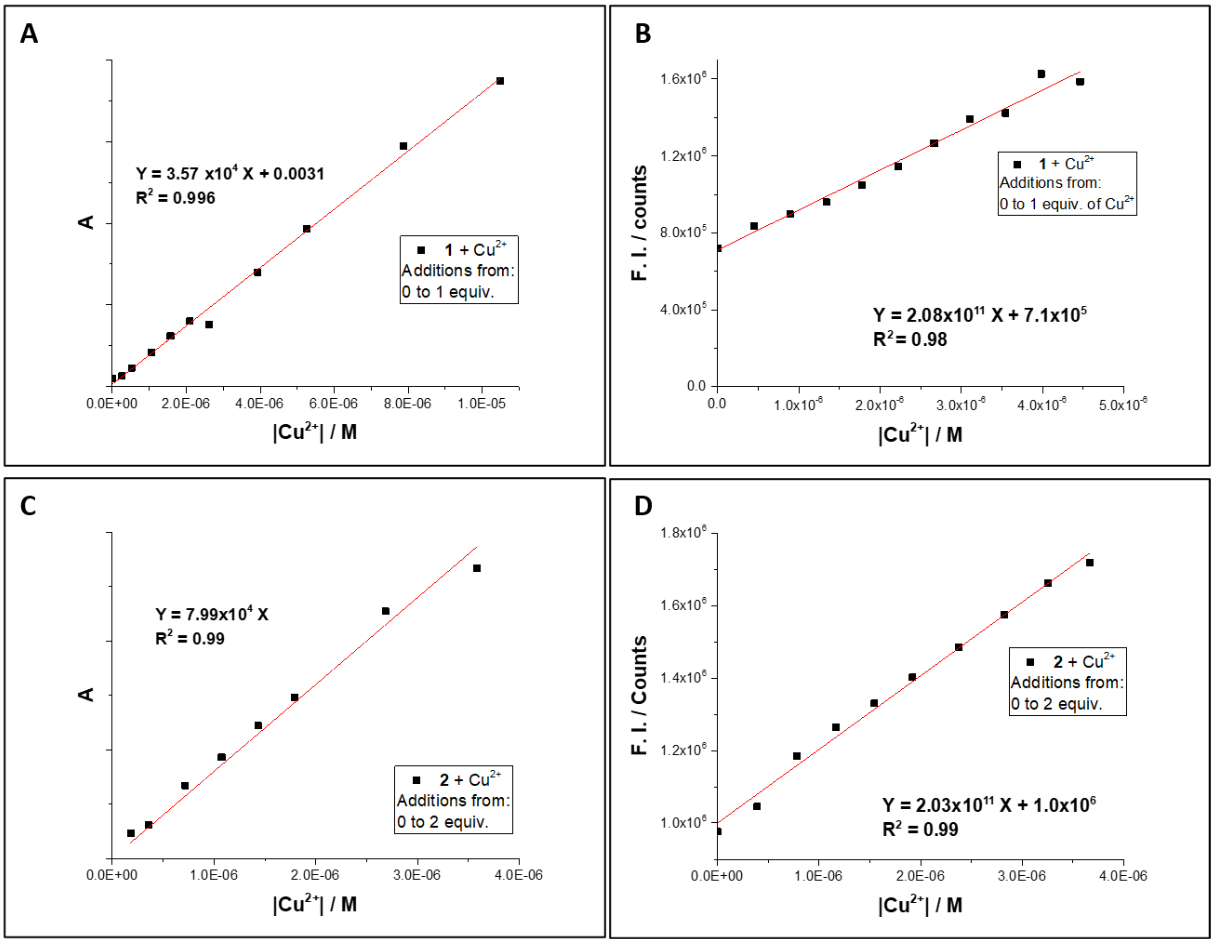

Figure S11. Absorption (**A** for **1** and **C** for **2**) and fluorescence (**B** for **1** and **D** for **2**) spectra of chemosensors **1** and **2** with increasing concentration of Cu^{2+}.

Absorbance and fluorescence measurements of chemosensors 1+Cu^{2+} and 2+Cu^{2+} in the presence of Li^{+}

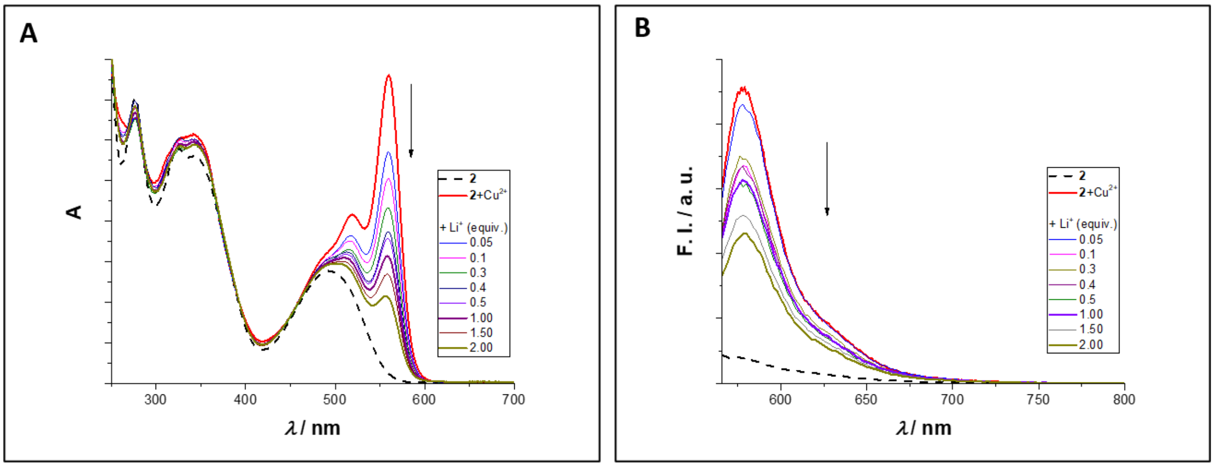

Figure S12. Absorbance (**A**) and fluorescence (**B**, using λ_{exc} = 560 nm) spectra of chemosensor **2**+Cu^{2+} with increasing concentrations of Li^{+} (up to 2 equiv.).

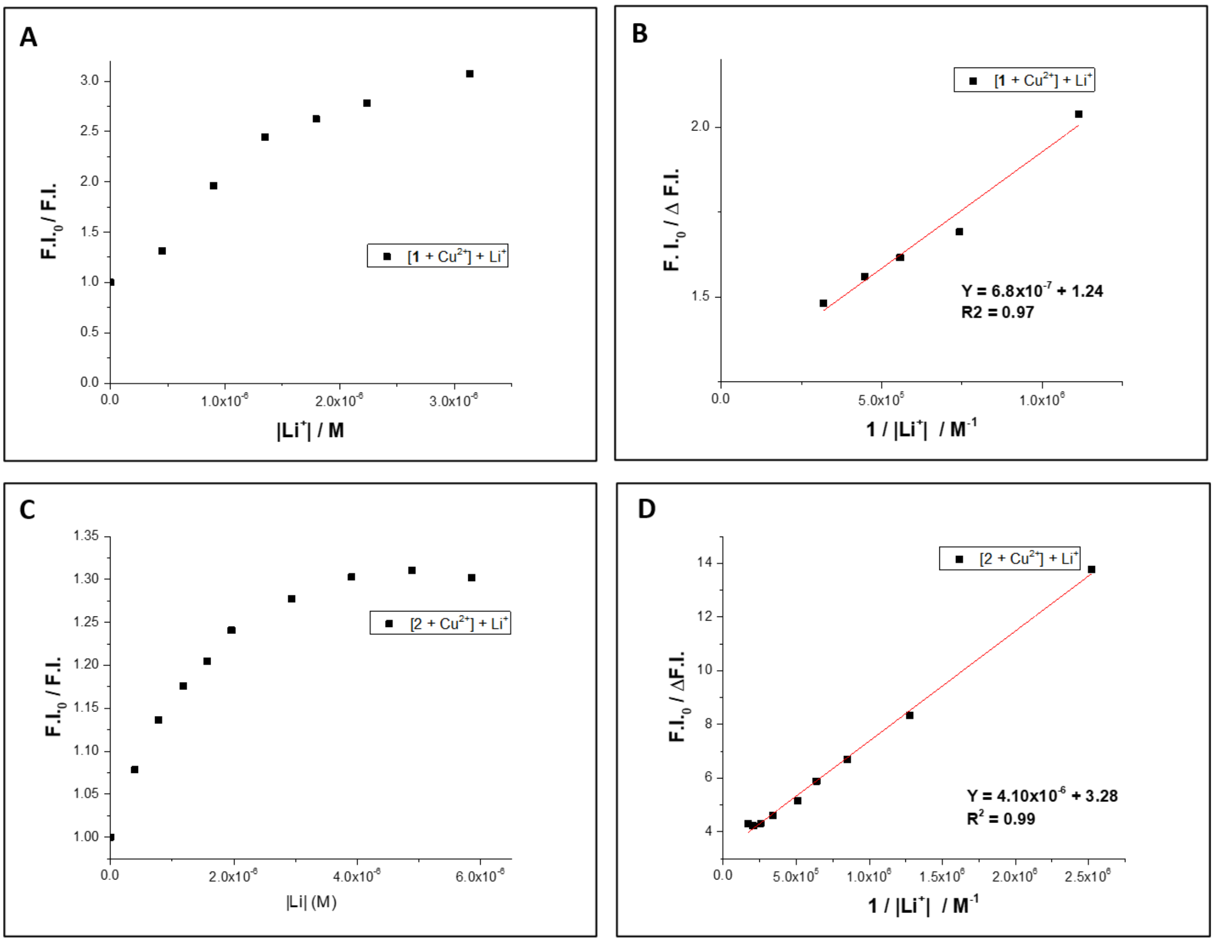

Figure S13. Stern-Volmer graphical representations for **1**+Cu^{2+} (**A** and **C** – modified version) and **2**+Cu^{2+} (**B** and **D** – modified version) in the presence of Li^{+}.

Absorbance and fluorescence measurements of chemosensor 2+Cu^{2+} in the presence of Li(CH_3COO) and LiBr salts

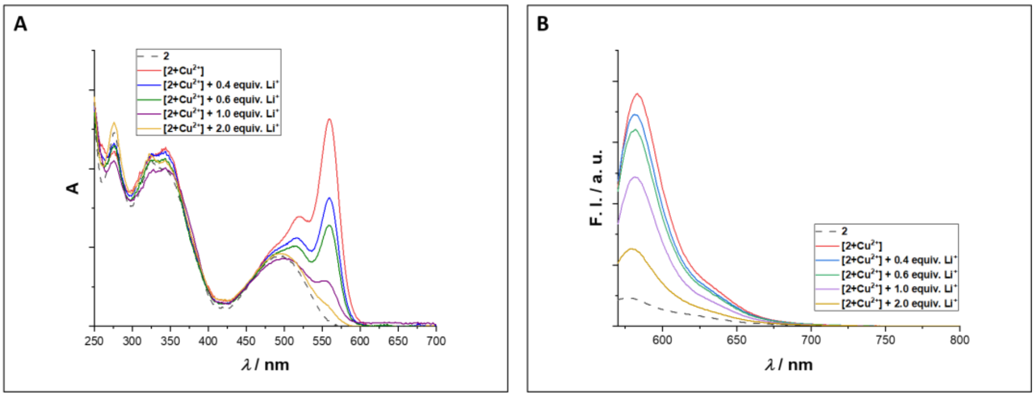

Figure S14. Absorbance (**A**) and fluorescence (**B**, using λ_{exc} = 560 nm) spectra of chemosensor **2**+Cu^{2+} with increasing concentrations of Li^+ (up to 2 equiv.), using Li(CH_3COO) salt.

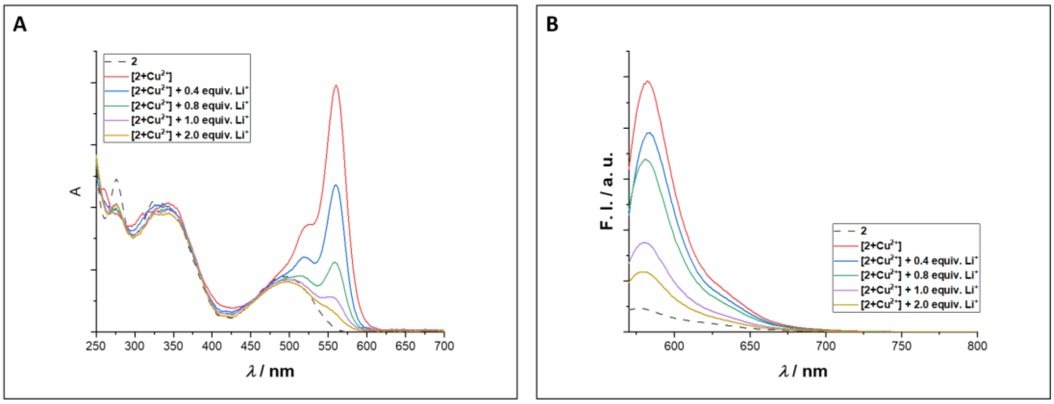

Figure S15. Absorbance (**A**) and fluorescence (**B**, using λ_{exc} = 560 nm) spectra of chemosensor **2**+Cu^{2+} with increasing concentrations of Li^+ (up to 2 equiv.), using LiBr salt.

Absorbance and fluorescence measurements of chemosensors 1+Cu^{2+} and 2+Cu^{2+} in the presence of CN^-

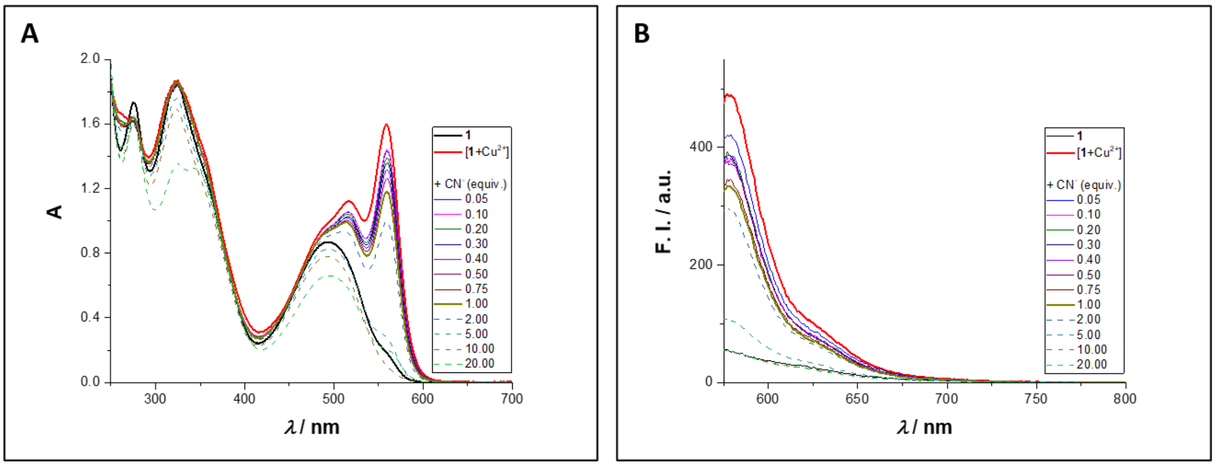

Figure S16. Absorbance (A) and fluorescence (B, using λ_{exc} = 560 nm) spectra of chemosensor **1**+Cu^{2+} with increasing concentrations of CN^- (up to 20 equiv.).

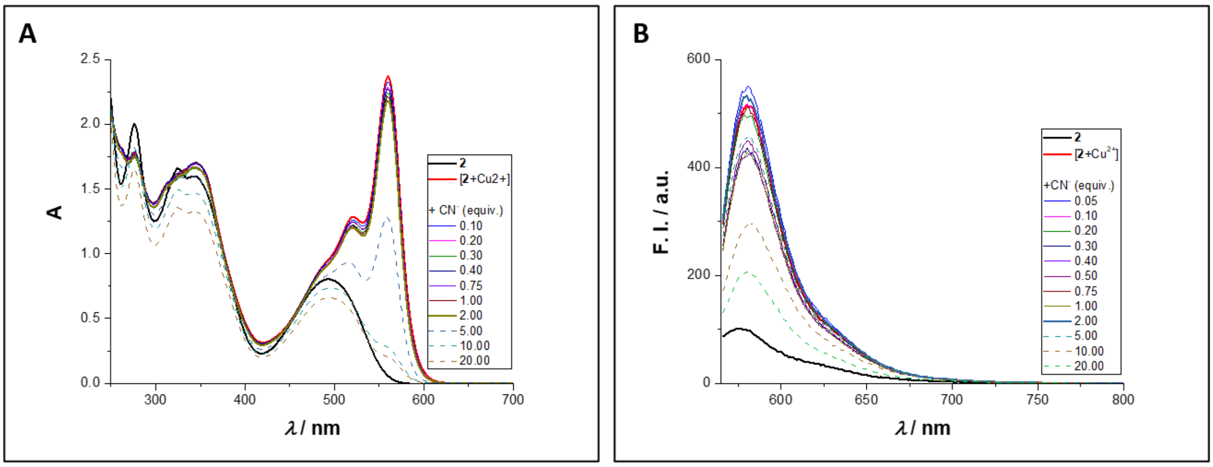

Figure S17. Absorbance (**A**) and fluorescence (**B**, using λ_{exc} = 560 nm) spectra of chemosensor **2**+Cu^{2+} with increasing concentrations of CN^- (up to 20 equiv.).

The results showed that upon addition of CN^- (up to 1 equiv.) to a solution of **1**+Cu^{2+}, a small decrease in the absorbance band at 560 nm and in the emission intensity at 577 nm was observed, around 30% (Figure S17). This is not the case for **2**+Cu^{2+} where the decrease is not relevant when 2 equiv. of CN^- are added (Figure S17). Further increase in CN^- up to 20 equiv. reveal a more significative decrease both in absorption and emission for both **1**+Cu^{2+} and **2**+Cu^{2+}, however the decrease is, in general, not linearly correlated with the increase in CN^- concentration. For both chemosensors, the addition of 5 equiv. or more of CN^- concentration also caused the decrease in the absorption band at 493 nm.

Stern-Volmer graphical representations of chemosensor 1+Cu^{2+} in the presence of CN^-

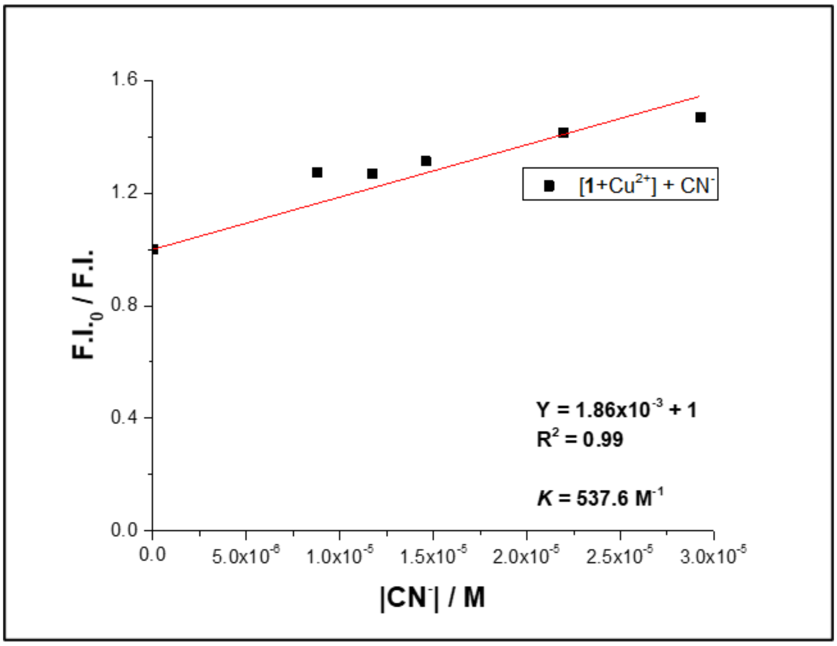

Figure S18. Stern-Volmer graphical representation for the complex **1**+Cu^{2+} in the presence of CN^-.
